# The Bicarbonate Transporter (MoAE4) Localized on Both Cytomembrane and Tonoplast Promotes Pathogenesis in *Magnaporthe oryzae*

**DOI:** 10.3390/jof7110955

**Published:** 2021-11-11

**Authors:** Yuejia Dang, Yi Wei, Penghui Zhang, Xinchun Liu, Xinrui Li, Shaowei Wang, Hao Liang, Shi-Hong Zhang

**Affiliations:** 1 College of Plant Protection, Shenyang Agricultural University, Shenyang 110866, China; dangyj@syau.edu.cn (Y.D.); wyziyu@syau.edu.cn (Y.W.); 2 Center for Extreme-Environmental Microorganisms, Shenyang Agricultural University, Shenyang 110866, China; 3 College of Plant Sciences, Jilin University, Changchun 130062, China; zhangph18@mails.jlu.edu.cn (P.Z.); lxc18@mails.jlu.edu.cn (X.L.); lixr19@mails.jlu.edu.cn (X.L.); wangsw15@mails.jlu.edu.cn (S.W.); lianghao8218@mails.jlu.edu.cn (H.L.)

**Keywords:** anion exchange protein 4 (AE4), HCO_3_^−^ transporter, tonoplast, pathogenicity, *Magnaporthe oryzae*

## Abstract

Bicarbonate (HCO_3_^−^) transporter family including the anion exchanger (AE) group is involved in multiple physiological processes through regulating acid-base homeostasis. HCO_3_^−^ transporters have been extensively studied in mammals, but fungal homologues of AE are poorly understood. Here, we characterized the AE group member (MoAE4) in *Magnaporthe oryzae*. MoAE4 exhibits more sequence and structure homologies with the reported AE4 and BOR1 proteins. In addition to the common sublocalization on cytomembrane, MoAE4 also localizes on tonoplast. Yeast complementation verified that MoAE4 rescues boron sensitivity and endows NaHCO_3_ tolerance in the *BOR1* deleted yeast. *MoAE4* gene is bicarbonate induced in *M. oryzae*; and loss of *MoAE4* (Δ*MoAE4*) resulted in mycelial growth inhibited by NaHCO_3_. Lucigenin fluorescence quenching assay confirmed that Δ*MoAE4* accumulated less HCO_3_^−^ in vacuole and more HCO_3_^−^ in cytosol, revealing a real role of MoAE4 in bicarbonate transport. Δ*MoAE4* was defective in conidiation, appressorium formation, and pathogenicity. More H_2_O_2_ was detected to be accumulated in Δ*MoAE4* mycelia and infected rice cells. Summarily, our data delineate a cytomembrane and tonoplast located HCO_3_^−^ transporter, which is required for development and pathogenicity in *M. oryzae*, and revealing a potential drug target for blast disease control.

## 1. Introduction

The bicarbonate anion (HCO_3_^−^)-transporter family, also known as the SLC4 (solute carrier 4) transporter family, functions to transport HCO_3_^−^ across the plasma membrane and in the maintenance of intracellular pH value. HCO_3_^−^ transporter proteins have been extensively studied in mammalians and invertebrates. In mammals there are 14 genes which encode proteins with bicarbonate transport activity [1]. According to the physiological activity, bicarbonate transporters can be classed into three major groups: Cl^−^/HCO_3_^−^ exchangers (AEs), Na^+^/HCO_3_^−^ cotransporters (NBCs), and Na^+^ dependent Cl^−^/HCO_3_^−^ exchangers (NDCBEs). The Cl^−^/HCO_3_^−^ exchangers AE1-3 are about 53–56% identical to one another at the amino-acid level. The electrogenic Na^+^/HCO_3_^−^ cotransporters NBCe1 and NBCe2 are about 28–34% identical to the AEs. The electroneutral Na^+^/HCO_3_^−^ transporters NBCn1, NDCBE, and NBCn2 are about 30–34% identical to the AEs, and about 39–50% identical to the electrogenic NBCs. Therefore, the deduced amino acid sequences of HCO_3_^−^ transporter proteins show a high degree of similarity to anion exchangers [2].

HCO_3_^−^ transporters share many common features in membrane topology, glycosylation, and inhibition by stilbene disulfonate inhibitors; but they are different in some ways such as the nature of transport activity and the subsidiary ions carried [1,2]. All function by an electroneutral mechanism, exchanging Cl^−^ for HCO_3_^−^ across the plasma membrane, driven by the respective gradients of the transport substrates [1].

The three identified anion exchangers (AE1-3) mediate the electroneutral exchange of one monovalent anion for another across the plasma membrane. The well characterized anion exchanger 1 (AE1), the first bicarbonate transporter cloned and sequenced Cl^−^/HCO_3_^−^ exchanger, is the erythrocyte band 3 glycoprotein that contains a membrane domain responsible for transport function. The crystal structure of the AE1 anion exchanger domain reveals a transport mechanism with which to understand the many mutations in the protein that lead to diseases [3,4].

The AE4 (SLC4A9) was originally reported to facilitate Cl^−^/HCO_3_^−^ exchange [5,6]. Based on the strong phylogenetic clustering of AE4 with reported Na^+^/HCO_3_^−^ co-transporters, AE4 was regarded as a Na^+^/HCO_3_^−^ co-transporter, not a Cl^−^/HCO_3_^−^ exchanger [2]. Native AE4 activity in mouse salivary gland acinar cells supports Na^+^-dependent Cl^−^/HCO_3_^−^ exchange that is comparable with that obtained upon heterologous expression of mouse AE4 and human AE4 in CHO-K1 cells. Particularly, AE4 mediates Cl^−^/HCO_3_^−^ exchange activity in the presence of K^+^ as well as Cs^+^, Li^+^, and Rb^+^ [7,8,9].

The AE4 gene is conserved in a variety of species. Homologous sequences and crystal structures of HCO_3_^−^ transporters have been identified not only in mammals, but also in fungi and plants [10]. The budding yeast *Saccharomyces cerevisiae* genome harbors the YNL275w gene (ScAE4), which showed some sequence identity to band 3 [11]. The YNL275w is transcribed at an extremely low level and not induced in response to nitrogen starvation. In addition, the YNL275w disruption mutant did not show any phenotype alteration under normal growth conditions. However, YNL275w homologue BOR1 is involved in tolerance to boric acid and the maintenance of the protoplasmic boron concentration [12,13], and BOR1 regulates a saturable uphill boron efflux, with characteristics consistent with a bicarbonate-independent exchange of extracellular H^+^ for intracellular H_3_BO_3_ [14]. Similarly, *Arabidopsis* YNL275w (*BOR1*) also supports plant boron tolerance [15]. Recently, several SLC family members such as SbtA (AN4904), SbtB (AN0218), and SB (AN2730) were characterized in *Aspergillus nidulans* [16]. SbtB functions as a BOR1 homologue; but SB appears to be a HCO_3_^−^ transporter.

The rice blast fungus *M. oryzae* is the causal agent of blast disease worldwide. Host infection is initiated by developed conidia, which occurs outside plant cells and involves conidium germination, tube elongation, appressorium maturation, and differentiation [17,18]. After penetration, successful development of invasive hyphae determines the severity of blast [19,20]. However, during invasive hyphae growth and development in the host plant, *M. oryzae* undergoes various harsher obstacles involving not only plant-derived passive and active resistance such as the accumulation of reactive oxygen species (ROS), antimicrobial compounds, and pathogenesis-related proteins for instance [21,22,23], but also *in planta* nitrogen starvation, high- HCO_3_^−^, and low-oxygen stresses [24,25,26]. To colonize the host successfully, *M. oryzae* must ensure a basic standard to survive these adverse environments. In this research, we biologically analyzed the *M. oryzae* AE4 homologue gene through creating deletion mutant and complementary strains. Biologic and molecular data reveal that MoAE4 is a cytomembrane and tonoplast localized HCO_3_^−^ transporter. Importantly, MoAE4 required for pathogenicity provides a new target for blast disease control.

## 2. Materials and Methods

### 2.1. Sequence Alignment Assays

The MoAE4 (MGG_15203) gene and amino acid sequences were acquired from the NCBI database (https://www.ncbi.nlm.nih.gov/, accessed on 25 March 2021). The protein tertiary and transmembrane structures were predicted using I-TASSER (https://zhanglab.ccmb.med.umich.edu/I-TASSER/, accessed on 25 March 2021), TMHMM Server v. 2.0 (http://www.cbs.dtu.dk/services/TMHMM/, accessed on 16 May 2021), and Softberry (http://www.softberry.com/berry.phtml, accessed on 16 May 2021). In addition, the amino acid sequence was aligned using the DNAMAN program, and the phylogenic tree was drawn using MEGA7.0.9 software.

### 2.2. Fungal Strains and Culture Conditions

*M. oryzae* strain JJ88 was used as the wild type. It was isolated and purified from *Oryza sativa* cultivar Jijing88, a variety that is widely planted in Jilin Province, China. All the fungal strains were cultured on complete media (CM) agar plates and maintained on paper filters at −20 °C (CM [10 g/L glucose, 2 g/L peptone, 1 g/L yeast extract, 1 g/L casamino acids, 0.1% (*V*/*V*) trace elements, 0.1% (*V*/*V*) vitamin supplement, 0.5 g/L MgSO_4_, 6 g/L NaNO_3_, 0.5 g/L KCl, and 1.5 g/L KH_2_PO_4_, pH 6.5]). For conidiation, the strains were inoculated on oatmeal–tomato agar medium (OMA) at 24 °C for 7 days in the dark [27]. The strains were grown continually for 3 days while illuminated under fluorescent lights after the aerial hyphae of the strains had been removed by washes with sterile distilled water.

*S. cerevisiae* BY4741 and the *ScBor1* deletion mutant strains (Invitrogen, Beijing, China) were used for functional complementation test. The yeast *S. cerevisiae* transformation was performed by the lithium acetate procedure. For yeast gene expression, YPB-ADHpt promoter and terminator regions of ADH1 gene in YPB1 was used [28]. All yeast strains were cultured according to Li et al. [29]. Δ*ScBor1* of *S. cerevisiae* was transferred in MoAE4 and MoACT, respectively. The mutants of Δ*ScBor1*, Δ*ScBor1*/*MoAE4*, Δ*ScBor1*/*MoACT*, and wild type were inoculated on to the Solid YPD medium Plates with 100 mM H_3_BO_3_ and 50 mM NaHCO_3_, respectively.

### 2.3. Assays for the Subcellular Localization of MoAE4

The localization of *MoAE4* in the wild type strain was observed by tagging it with the *Bgl* Ⅱ-*Spe*I sites of green fluorescent protein (GFP) of vector pCAMBIA1303 at its C-terminus. We generated transgenic strains expressing GFP-tagged *MoAE4* fusion gene in the wild type of *M. oryzae* (pCAMBIA1303-MoAE4:: GFP). Fluorescent microscopic observation was carried out by using hyphae (6d) and conidia (6d). To visualize the cytoplasmic membrane and vacuolar membrane, vegetative hyphae and conidia were treated with 2 μg/mL FM4-64 (AAT Bioquest, Sunnyvale, CA, USA) solution for 30–60 min before observed [30] under laser scanning confocal microscope (Olympus fluoview FV3000, Olympus, Tokyo, Japan).

### 2.4. Targeted Gene Deletion and Complementation

To generate the *MoAE4* replacement construct pXEH20, the upstream (1155 bp) and downstream (1195 bp) fragments of *MoAE4* were amplified using primers MoAE4-L-S/MoAE4-L-A and MoAE4-R-S/MoAE4-R-A, respectively. The resulting PCR products were cloned into the *Spe*I-*Kpn*I and *Xba*I-*Hind*III sites of vector pXEH2.0. The knockout vector was introduced into *Agrobacterium tumefaciens* strain AGL-1 and then transformed into the wild type *M. oryzae* using the *A. tumefaciens*-mediated transformation (ATMT) method as previously described [31]. Transformants were selected and cultured in 200 μg/mL hygromycin. The transformants were identified using PCR with primers HYG-S/HYG-A, MoAE4-LHYG-S/MoAE4-LHYG-A, and MoAE4-G-S/MoAE4-G-A.

The entire *MoAE4* sequence was amplified using a PCR technique with MoAE4-C-S/MoAE4-C-A and inserted into the hygromycin resistant vector pCAMBIA1303 for complementation into the mutant strain. The reconstructed pCAMBIA1303-*MoAE4* was transformed into the Δ*MoAE4* mutant strain and designated Δ*MoAE4*/*MoAE4*. The complemented strain was also confirmed by PCR with HYG-S/HYG-A and MoAE4-G-S/MoAE4-G-A.

To further verify the gene deletion and complementation, the expression of the wild type, Δ*MoAE4* mutant, and Δ*MoAE4*/*MoAE4* strains was amplified using qRT-PCR with qRT-MoAE4-S/qRT-MoAE4-A and Actin-S/Actin-A, and the strains were identified. The primers for gene deletion and complementation are listed in Table S2.

### 2.5. Quantitative Real-Time PCR (qRT-PCR)

The total RNA was isolated from mycelia that had been harvested from 5-day-old CM media using the TRIzol reagent (Invitrogen, Carlsbad, CA, USA). First strand cDNA was synthesized using an oligo (dT) primer from total RNA, which had been treated with DNase I. Subsequently, qRT-PCR was performed using an ABI7500 System (Applied Biosystems, Foster City, CA, USA) and SYBR Premix Ex Taq (TaKaRa, Dalian, China). The relative mRNA levels were calculated using the 2^−ΔΔCq^ (C_q_ = C_qgene_ − C_qactin_) method. The *M. oryzae* actin gene (MGG_03982.6) was utilized as a reference gene for normalization. Each sample was tested in three replicates in each experiment. The primer sequences used for qRT-PCR are shown in Table S2.

### 2.6. Assays for Conidial Production, Growth, and Development

The strains (wild type, Δ*MoAE4*, and Δ*MoAE4*/*MoAE4*) were cultured on PDA media to understand the effect on *MoAE4* conidial production, and the conidia were cultured on OMA media as previously described. A volume of 200 μL of a 1 × 10^5^/mL conidial solution was placed on OMA medium. After 3 days of cultivation at 28 °C, sterile water was added to remove the hyphae, and a piece of the culture medium was cut with a blade and placed on a glass slide. It was then placed in a moisturizing box and incubated at 28 °C. The piece was observed under a Nikon Eclipse 80i microscope at 6, 12, 24, and 48 h after it had been cut. The strains were then stained with lactophenol cotton blue to observe the conidiophore stalks and hyphae under a light microscope [32]. Additionally, the conidia were collected with 2 mL of sterile water after 3 days of culture on OMA media and counted with a hemocytometer. Each strain was repeated three times, and the experiment was conducted in triplicate.

Conidia of the wild type, Δ*MoAE4*, and Δ*MoAE4*/*MoAE4* were cultured on OMA media and collected to observe the germination of conidia and formation of appressoria. The conidial suspension was adjusted to 1 × 10^5^/mL and added drop wise to a hydrophobic cover slips under a microscope at 1, 2, 3, 4, 5, and 6 h. Each strain was repeated three times, and the experiment was conducted in triplicate.

### 2.7. Rice Sheath Penetration and Plant Infection Assays

To determine the pathogenicity of *MoAE4,* the wild type, Δ*MoAE4*, and Δ*MoAE4*/*MoAE4* strains were inoculated on OMA media to collect the conidia as previously described. The fourth leaf stage of rice seedlings (*Oryza sativa* cv. Lijiangxintuanheigu) was assayed for infection following the spraying of 2 mL of a conidial suspension (5 × 10^4^ conidia/mL in 0.2% gelatin). The inoculated plants were placed in the dark in a dew chamber for 24 h at 28 °C and then transferred to a growth chamber with a photoperiod of 16 h for 7 days.

Conidial suspensions (100 μL, 5 × 10^4^ conidia/mL) were injected into seedling leaf sheaths using a 1 mL syringe, and the inoculated plants were placed in a moist chamber as described previously. The formation of lesions and necrosis around the inoculation sites was examined when the injection-wounded leaves unfolded at different time points after the injection. The mean infectious hyphal (IH) growth rates and movement to the adjacent cells were determined from 100 germinated conidia per treatment at 12, 24, and 48 h post inoculation (hpi) and repeated in triplicate as previously described. The leaf sheaths were trimmed at the time points indicated and observed using a Nikon Eclipse 80i microscope. This experiment was performed with three independent replicates, and the representative results from one of these experiments are presented.

### 2.8. Assays for NaHCO_3_ Treatment

To illustrate the effect of different concentrations of NaHCO_3_ on the expression of *MoAE4* gene, wild type strains of *M. oryzae* were cultivated on PDA with 0, 12.5, 25, 37.5, 50, 62.5, and 75 mM NaHCO_3_ at 28 °C for 7 days.

To investigate the effects of sodium bicarbonate stress on the wild type, Δ*MoAE4*, and Δ*MoAE4*/*MoAE4* strains, each strain was cultured on PDA with NaHCO_3_ at final concentrations of 0, 12.5, 25, 37.5, 50, 62.5, and 75 mM at 28 °C for 7 days, and the diameters of fungal strains were photographed using a digital camera (EOS 800D, Canon, Inc., Tokyo, Japan) and measured after inoculation. Each assay was repeated three times independently for each strain, and the experiment was performed in triplicate. Further, the wild type of mycelium treated under different concentrations of NaHCO_3_ was collected for expression patterns of AE4.

### 2.9. Assays for HCO_3_^−^ Transport and Intracellular pH Measurements

To determine the relationship between *MoAE4* and HCO_3_^−^ transport, confocal microscope was performed with lucigenin (bis-N-methylacridinium nitrate) (MCE, Shanghai, China), a compound that is used as a chemiluminescent probe to indicate the presence of superoxide anion radicals in cells in alkaline conditions 28 [33,34]. The microscopy enabled the detection of direction of HCO_3_^−^ transport in the conidia and hyphae. First, conidia and hyphae from the strains (wild type, Δ*MoAE4*, and Δ*MoAE4*/*MoAE4*) were treated at 28 °C with 0.4 M mannitol in a solution of 50 mM NaHCO_3_ for 2 h, then washed and added 0.4 M mannitol to continue recovery at 28 °C for 2 h. A solution of only 0.4 M mannitol served as the control. The strains were incubated with 10 mM lucigenin and observed with a 470 nm fluorescence microscope.

The intracellular pH was measured using the dual-excitation ratio method with the pH sensitive dye 20,70-bis-(2-carboxyethyl)-5- (and-6)-carboxyfluorescein acetoxymethyl ester (BCECF-AM) (Mock, Sigma, Shanghai, China) to detect the wild type, Δ*MoAE4*, and Δ*MoAE4*/*MoAE4* strains treated under 50 mM NaHCO_3_ as previously described. The pH sensitive dye was excited at 460 and 488 nm using a digital fluorescence microscopy system, and the fluorescence emitted at 520 nm was detected. The ratios of background-corrected emission intensities (I488/I460) were transformed into intracellular pH [35,36].

### 2.10. H_2_O_2_ Treatment and Endogenous H_2_O_2_ Measurements

To investigate the effects of exogenous oxidative stress on the wild type, Δ*MoAE4*, and Δ*MoAE4*/*MoAE4* strains, each strain was cultured on CM agar that contained 2.5 mM or 5 mM H_2_O_2_ for 7 days at 28 °C.

The H_2_O_2_ content was determined as previously described for plants [37]. Hydrogen peroxide (H_2_O_2_) was extracted by homogenizing 3 g of mycelia from the wild type, Δ*MoAE4*, and Δ*MoAE4*/*MoAE4* strains in 6 mL of cold acetone. The homogenate was then centrifuged at 3500× *g* for 5 min at room temperature, and the resulting supernatant was designated as the sample extract. Next, 0.1 mL of titanium reagent (5% [*w*/*v*] titanic sulfate in concentrated H_2_SO_4_) was added to 1 mL of the sample extract, followed by the addition of 0.2 mL of strong aqueous ammonia to precipitate the peroxide-titanium complex. The precipitated sample was centrifuged at 3000× *g* for 10 min at room temperature; the supernatant was discarded, and the precipitate was then solubilized in 5 mL of 2 M H_2_SO_4_. The absorbance of the samples was determined at 415 nm against a blank of 2 M H_2_SO_4_. The H_2_O_2_ concentration in the samples was determined by comparing the absorbance against a standard curve of a 0–5 mM titanium-H_2_O_2_ complex that was prepared according to Cui et al. [38].

The production of H_2_O_2_ was monitored by staining with 3,3′-diaminobenzidine (DAB) as an assay [39]. The hyphae of the wild type, Δ*MoAE4*, and Δ*MoAE4*/*MoAE4* strains were cultured in CM media for 5 days and then incubated in the dark in a 1 mg/mL solution of DAB at room temperature for 8 h. The samples were washed with sterile water and observed under a Nikon light microscope. This experiment was performed in triplicate and repeated three times for each strain. Similarly, leaf sheath cells of rice infected by wild type, mutant, and complementation strains were stained DAB at 36 hpi.

The conidia of the wild type, Δ*MoAE4*, and Δ*MoAE4*/*MoAE4* strains were extracted with DMSO [40].

### 2.11. Statistical Analysis

All the experiments were performed at least three times. The mean ± SD of the strain diameter, germination rate, and relative expression were determined using SPSS Statistics 22 (IBM, Inc., Armonk, NY, USA). Error bars represent the standard deviation. * indicates a statistically significant difference at *p* < 0.05. ** indicates a highly significant difference at *p* < 0.01. *** indicates a highly significant difference at *p* < 0.001. **** indicates a highly significant difference at *p* < 0.0001.

## 3. Results

### 3.1. The Bicarbonate Transporter AE4 Homologue in M. oryzae

Homologous sequences of AE4 proteins have been reported in a variety of species. Based on the conserved amino acid sequences of several reported AE4 proteins, a single homolog of AE4 (MGG_15203) was searched in the *M.* *oryzae* genome (http://fungalgenomics.ca/wiki/Fungal_Genomes, accessed on 25 March 2021). The *M.* *oryzae AE4*, termed as *MoAE4*, with a length of 2169 bp open reading frame, encodes a protein of 701 amino acids. The protein sequence alignments delineated the 10 transmembrane-spanning domains in the MoAE4 gene (Figure S1A,B).

**Figure 1:**
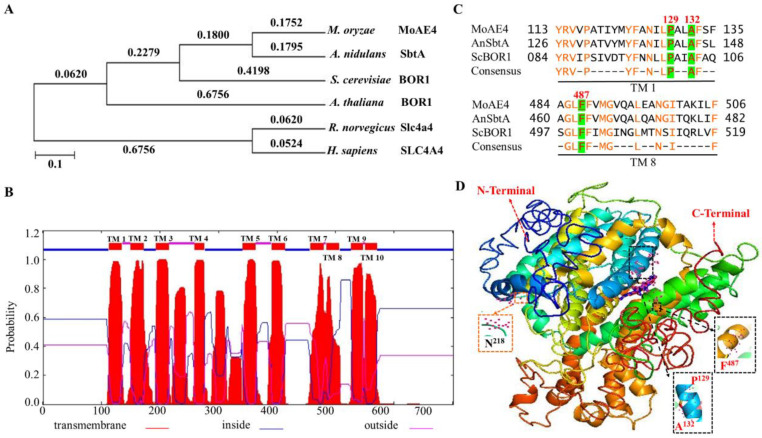
Structure of the MoAE4 and phylogenetic analysis. (**A**) Phylogenetic tree. It was constructed with reported anion exchange protein 4 homologs from *M. oryzae*, *A. nidulans*, *S. cerevisiae*, *A.* *thaliana*, *R. norvegicus*, *H. sapiens*, indicating that MoAE4 has a relatively close relationship with the fungal group. (**B**) TMHMM posterior probabilities for WEBSEQUENCE (on-line analysis). A schematic diagram of the MoAE4 protein transmembrane domain of *M. oryzae* is shown indicating the 10 transmembrane domains and marked the amino acid inside and outside the membrane. (**C**) Sequence alignment. The transmembrane domain 1 and 8 of MoAE4 was compared with closely related to the fungal group *A. nidulans* SbtA gene and *S. cerevisiae* BOR1 gene. (**D**) Tertiary structure (3-D). The structure was predicted using the web-based server I-TASSER that shows the ligand binding site residues of transmembrane domain P^129^, A^132^, and F^487^. The active site residue is N^218^.

The phylogenic tree indicated that *MoAE4* was closely related to the fungal group (Figure 1A), sharing 75.6% identity with *A. nidulans* SbtA gene, and 57.5% identity with *S. cerevisiae* YNL275w gene. The transmembrane-spanning domains and tertiary (3D) structures of MoAE4 were predicted with the web-based TMHMM Server v.2.0 (http://www.cbs.dtu.dk/services/TMHMM/, accessed on 16 May 2021) and I-TASSER (http://zhanglab.ccmb.med.umich.edu/I-TASSER/, accessed on 25 March 2021). Both the N-terminal and C-terminal ends are membrane inside (Figure 1B and Figure S1B), which are responsible for transport activity and transmembrane domain anchoring [41]. The two regions TM1 and TM8, together with the N-terminal and C-terminal ends, form the so-called gate domain and core domain. The three conserved ligand sites (P^129^, A^132^, and F^487^) and the active site N^218^ harbors in the C-terminal end (core domain) (Figure 1C,D and Figure S1C).

According to the 3D structures (Figure 1D and Figure S1C), the conformations with 10 TM helices of MoAE4 were characterized by two inverted repeats that are intertwined to form both core and gate domains, which appears to be similar with that of AE1 or BOR1 [3,10]. The homologous protein sequences and the typical domain patterns reveal MoAE4 is a member of the HCO_3_^−^ transporter AE4 group.

### 3.2. MoAE4 Localizes on Cytomembrane and Tonoplast and Functions in Yeast

**Figure 2:**
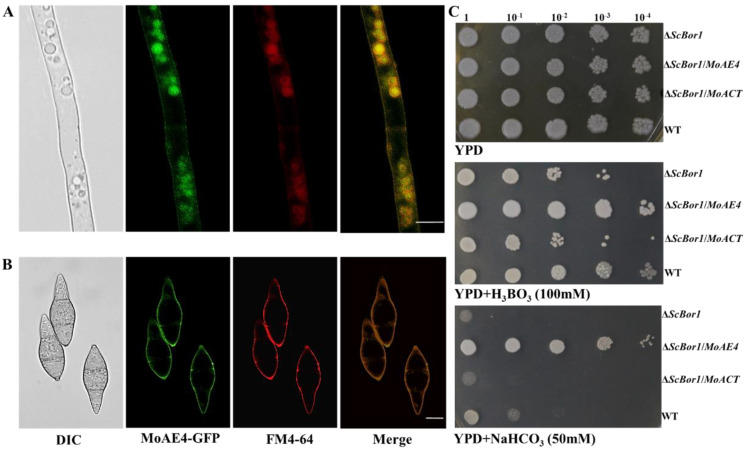
MoAE4 subcellular localization and functions in yeast. (**A**) Subcellular localization in hyphae (6d). Green fluorescence signals of the MoAE4–GFP protein of 6–day–old hyphae was examined by confocal microscopy and co–localized with FM4–64 on cytomembrane and tonoplast. Scale bar = 10 µm. (**B**) Subcellular localization in conidia. A strong green fluorescence signal of the MoAE4–GFP protein co–localized with FM4–64 red fluorescence was detected on the cytoplasmic membrane in conidia. Scale bar = 10 µm. (**C**) Functional complementation of MoAE4 for ScBor1 in *S. cerevisiae*. In total, 10 μL droplets containing the indicated concentration of yeast cells were inoculated on to the Solid YPD medium Plates (100 mM H_3_BO_3_ and 50 mM NaHCO_3_ added, respectively). The *MoAE4* gene could functionally reverse the defect of the Δ*ScBor1* mutant in boric acid and NaHCO_3_ tolerance. Representative plates were photographed 3 days post-inoculation.

Most HCO_3_^−^ transporters function on cytoplasm membrane [16]. To test where MoAE4 occurred, we generated transgenic strains expressing GFP-tagged *MoAE4* fusion gene in the wild type of *M. oryzae* (Figure S2A). Fluorescent microscopic observation was carried out by using hyphae (6d) and conidia. The lipophilic dye (FM™ 4-64 Dye, AAT Bioquest, USA) was used for observing the cytoplasmic membrane and vacuolar membrane. A strong green fluorescence signal of the MoAE4-GFP protein co-localized with FM4-64 red fluorescence was detected on the cytoplasmic membrane in young hyphae and conidia (Figure 2A,B); interestingly, a strong co-localized yellow fluorescence signal was also detected on the vacuole membrane in hyphae (Figure 2A,B). By comparison, in the wild type or untransformed strains, the background green fluorescence was too weak to be detected. This result suggests that MoAE4 was targeted to cytomembrane and tonoplast.

Yeast bicarbonate transporters are boric acid tolerant [12,13,14]. Based on the *ScBor1*/*AE4* deletion mutant (Δ*ScBor1*), the complementary yeast strains were created by using *MoAE4*. As a result, the *MoAE4* gene could functionally reverse the defect of the Δ*ScBor1* mutant in boric acid tolerance (Figure 2C), suggesting *MoAE4* functions as yeast Bor1. Different from the yeast BOR1, MoAE4 also endowed the NaHCO_3_ tolerance in the mutant strains (Figure 2C), implying MoAE4 may be a HCO_3_^−^ transporter.

### 3.3. MoAE4 Transports Cytosolic HCO_3_^−^ to Vacuole and Cell outside

**Figure 3:**
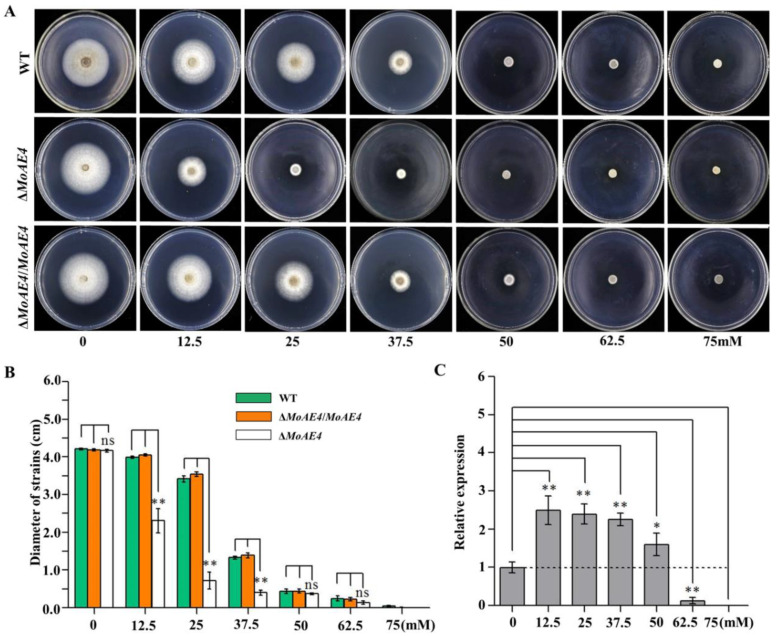
NaHCO_3_ stress assay of the wild type and created strains and expression patterns of MoAE4. (**A**) NaHCO_3_ stress assay. Δ*MoAE4* strains were more sensitive to NaHCO_3_ stress than the wild-type strains. The strains were cultured in PDA media at 28 °C under 0–75 mM different concentrations of NaHCO_3_ and representative colonies were photographed 7 days post-inoculation. (**B**) The colonies diameter of the wild type, the Δ*MoAE4* and Δ*MoAE4*/*MoAE4* mutant strains following treatments under different concentrations of NaHCO_3_. (**C**) Transcription abundance of MoAE4 under different concentrations of NaHCO_3_. Data represent the means ± standard deviation (SD) from three independent experiments in which triplicate plates were examined for each strain in each experiment. ns *p* > 0.05. * *p* < 0.05. ** *p* < 0.01.

To identify the function of MoAE4 in response to NaHCO_3_, *M. oryzae* was cultivated under NaHCO_3_ stress conditions. By using the knockout mutant strain of *MoAE4* (Δ*MoAE4*) and the complemented strain (Δ*MoAE4*/*MoAE4*) (Figure S2B–D), the growth of the tested strains was assessed. When cultivated on complete media (CM) plates at 25 °C without NaHCO_3_, both the Δ*MoAE4* and Δ*MoAE4*/*MoAE4* strains grew at a rate similar to that of the wild type, and their colony morphologies exhibited little difference (Figure 3A,B). When subjected with NaHCO_3_ of different concentrations, all strains including the wild type were repressed in mycelial growth under NaHCO_3_ stress. Impressively, Δ*MoAE4* almost stopped growing at 25 mM of NaHCO_3_, but the wild type and complementary strains were capable of growing even at the concentration of 37.5 mM (Figure 3A,B), suggesting a role of Δ*MoAE4* in tolerance to NaHCO_3_. The expression patterns in response to NaHCO_3_ treatment also illustrated this point (Figure 3C).

**Figure 4:**
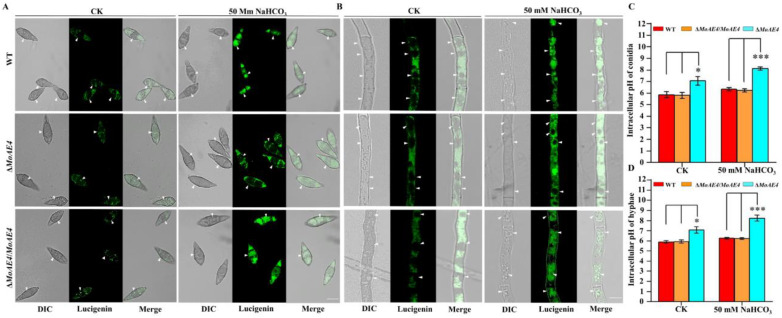
The fluorescence observations and intracellular pH value of the wild type, the Δ*MoAE4* and Δ*MoAE4*/*MoAE4* mutant strains. (**A**) The green fluorescence signals in hyphae. The light (**left**), fluorescence confocal (**middle**), and merge (**right**) microscope observations of strains hypha. Bar = 10 µm. (**B**) The green fluorescence signals in conidia. The light (**left**), fluorescence confocal (**middle**), and merge (**right**) microscope observations of strains connidia. Bar = 10 µm. (**C**) The intracellular pH value of conidia. Δ*MoAE4* strains were obviously higher than the wild-type strains under 50 mM sodium bicarbonate. (**D**) The intracellular pH value of the strains in hyphae. Under 50 mM sodium bicarbonate conditions, the intracellular pH of Δ*MoAE4* strains remained high in hyphae. The data represent means ± standard deviations (SD) of three experiments. * *p* < 0.05. *** *p* < 0.001.

To determine the HCO_3_^−^ transport activity of *MoAE4*, lucigenin (bis-N-methylacridinium nitrate), a chemiluminescent probe, was used as to detect the existence of anion radicals in cells under alkaline conditions [33,34]. After treated with NaHCO_3_ solution (50 mM), the tested strains incubated with 10 mM lucigenin were observed with a 470 nm fluorescence microscope. As expected, lucigenin probed HCO_3_^−^ with green fluorescence signals was accumulated in vacuoles of the wild type and Δ*MoAE4*/*MoAE4* strains; however, in Δ*MoAE4*, green fluorescence signals were only detected in cytoplasm (Figure 4A,B). Accordingly, intracellular pH value increased significantly in Δ*MoAE4* (Figure 4C,D and Figure S3). These results indicated that *MoAE4* functions as a *bona fide* HCO_3_^−^ transporter.

### 3.4. MoAE4 Is Important for Conidiation and Appressorium Development

**Figure 5:**
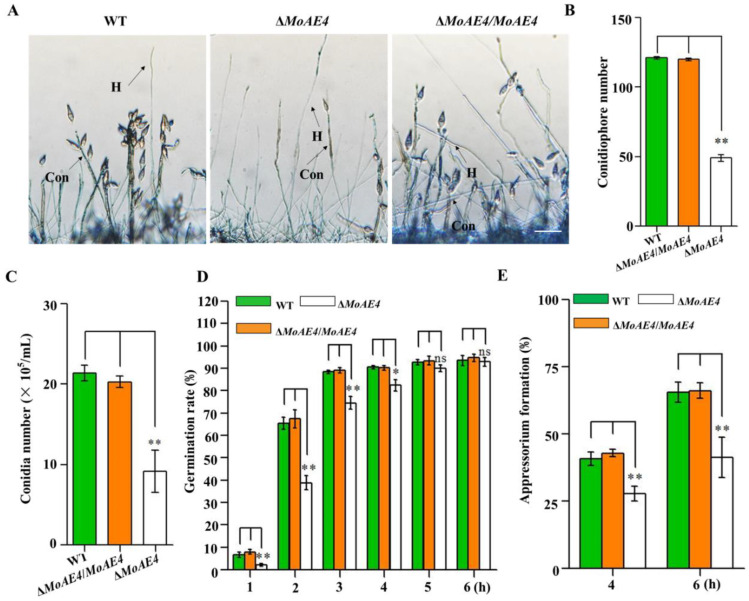
Conidium and appressorium development analysis of the wild type and created strains. (**A**) Conidiophores stained with lactophenol cotton blue. The conidiophores of the wild type, the Δ*MoAE4* and Δ*MoAE4*/*MoAE4* strains induced for 48 h were stained with lactophenol cotton blue, and observed and counted under a light microscope at room temperature. The hyphae are stained blue, whereas the conidiophore stalks are in gray. Bar = 50 μm. Con, conidiophores; H, hyphae. (**B**) Statistical analysis of the conidiophores number of the wild type, the Δ*MoAE4* and Δ*MoAE4*/*MoAE4* mutant strains. (**C**) Statistical analysis of conidial production in the strains. The conidia were harvested from the 3-day-old mycelium grown on OMA media, and counted using a hemocytometer for all the three strains. (**D**) Conidial germination rate. Conidial germination was measured on a hydrophobic cover slips and was calculated under the microscope at 1, 2, 3, 4, 5, and 6 h post inoculation. (**E**) Appressorial formation rate. Appressorial formation was measured on a hydrophobic cover slips and was calculated under the microscope at 4 and 6 h per inoculation. The analysis was performed using an independent samples *t*-test. ns *p* > 0.05. * *p* < 0.05. ** *p* < 0.01. Error bars indicate the mean ± SD from three independent experiments.

Conidiation and appressorium formation were analyzed among the Δ*MoAE4*, Δ*MoAE4*/*MoAE4*, and wild type strains. The sparse conidiophores with less conidia were observed in the deletion mutant Δ*MoAE4*, however, both the Δ*MoAE4*/*MoAE4* and wild type produced thick conidiophores and more conidia (Figure 5A–C and Figure S4).

The conidium germination rate of all strains including the wild type was similar at 4–6 h, although Δ*MoAE4* appeared to be a little slow in conidium germination at 1–4 h (Figure 5D and Figure S4B,C). In terms of appressorium formation, Δ*MoAE4*/*MoAE4* had the formation rate similar as the wild type did; but Δ*MoAE4* was severely affected (Figure 5E). As conidia and appressoria are essential factors for disease cycle and infection, *MoAE4* is proposed to be involved in pathogenesis.

### 3.5. Requirement of MoAE4 for Pathogenicity in M. oryzae

**Figure 6:**
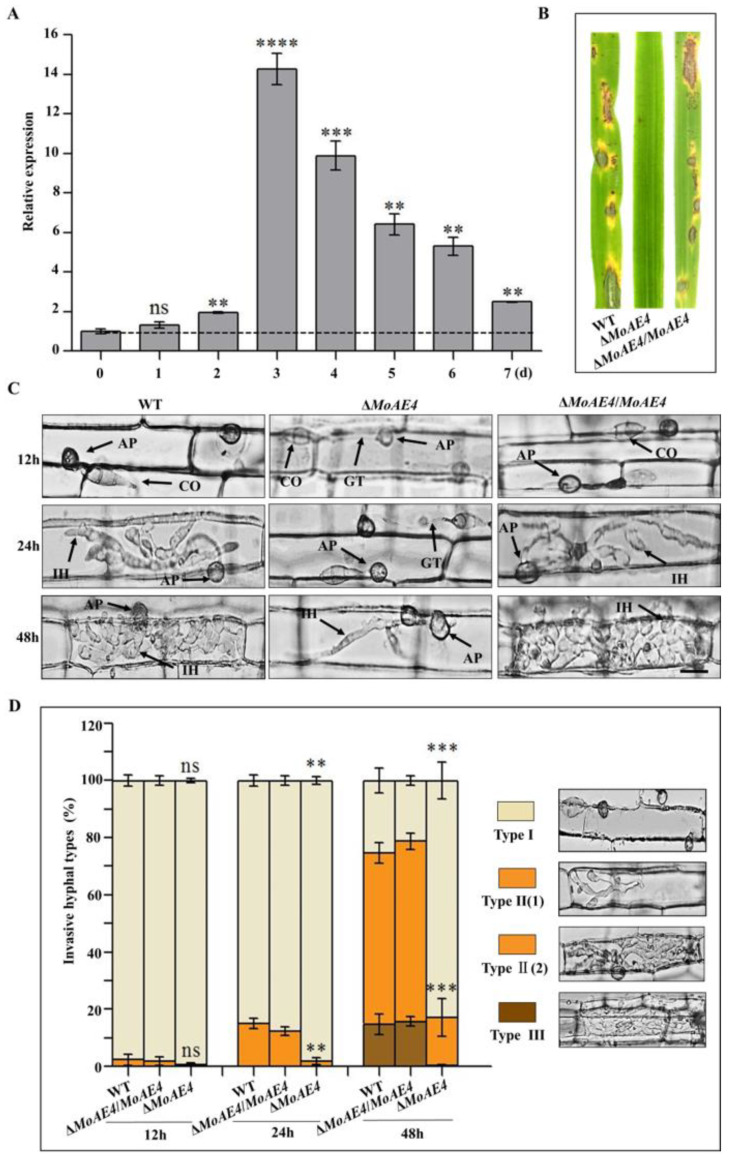
Pathogenesis analysis of the wild type and created strains. (**A**) Transcription abundance of *MoAE4* during disease development. (**B**) Spray-inoculation assay. (**C**) Rice leaf sheath infection assay. Scale bar = 10 μm. IH, infectious hyphae; CO, Conidium; GT, Germination tube; AP, appressorium. (**D**) The infection rate was calculated according to the number of type I to type III events. The infection status of more than 100 germinated conidia per leaf sheath was scored at 12, 24, and 48 h post inoculation. Type I, conidia with mature appressoria; Type II, primary hyphae formed, infectious hyphae extended and branched in one cell; Type III, infectious hyphae crossing to neighboring cells. Values represent the averages of five measurements ± standard deviation. The statistical analysis was performed using a one-way ANOVA with Tukey’s multiple comparison test. The averages were taken from the quadruplicate analysis. Values are based on three biological samples and error bars indicate SD, ns *p* > 0.05. ** *p* < 0.01; *** *p* < 0.001. **** *p* < 0.0001.

In order to characterize the function of MoAE4 in pathogenic development, pathogenicity assays were carried out using conidia collected from Δ*MoAE4*, Δ*MoAE4*/*MoAE4*, and the wild type. When intact susceptible rice seedlings were spraying-inoculated, at 7 days post inoculation (dpi), some acute expansive disease lesions were observed in rice leaves by the wild type and Δ*MoAE4*/*MoAE4*; but no lesions were formed in rice leaves by the Δ*MoAE4* (Figure 6). Similarly, when drop-inoculation was assayed, only the wild type and complementary strains still showed pathogenicity.

Leaf sheath infection assays were performed to examine the infection effects of the *MoAE4* in rice host. At 12 hpi, most mature (black) appressoria have been formed in the wild type and Δ*MoAE4*/*MoAE4*, but less in Δ*MoAE4*. At 24 hpi, invasive hyphae of the wild type and Δ*MoAE4*/*MoAE4* commenced to branch in rice cells, but the primary infectious hyphae were just formed in Δ*MoAE4*. At 48 hpi, the majority of invasive hyphae of the wild-type and Δ*MoAE4*/*MoAE4* branched and started entering neighboring cells, but the Δ*MoAE4* strains did not due to the defects in appressorium formation (Figure 6C,D).

To decipher the exact action of *MoAE4* during pathogenic development, we defined the three types of infection hyphae according to their developmental morphologies. Then we quantified the proportion of the three types of infection hyphae based on 100 germinated conidia in the inoculated leaf sheath (Figure 6D). As a result, at 48 hpi more than 60% of inoculated conidia from Δ*MoAE4*/*MoAE4* and wild type formed branched infectious hyphae in one cell, of which about 20% extended to neighboring cells of rice (type II and III), suggesting the requirement of *MoAE4* in pathogenesis.

### 3.6. MoAE4 Is Important for H_2_O_2_ Tolerance and Clearance Inside or Outside Cells

**Figure 7:**
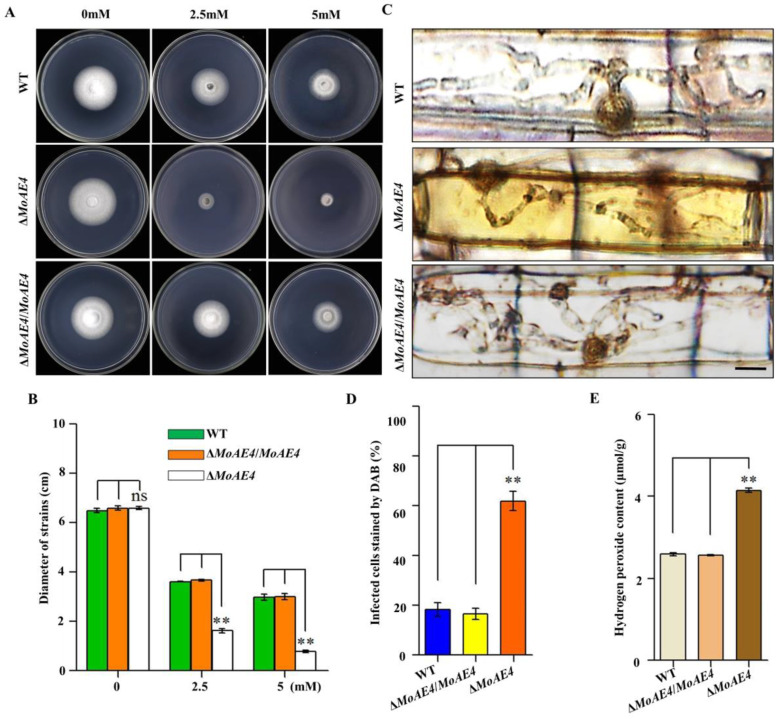
Comparison of oxidative stress, DAB staining, and endogenous H_2_O_2_ among the wild type, the Δ*MoAE4*, and Δ*MoAE4*/*MoAE4* strains. (**A**) H_2_O_2_ stress assay. The strains were cultured in CM media for 7 days at 28 °C with 2.5 or 5 mM H_2_O_2_. (**B**) The colonies diameter of the wild type, Δ*MoAE4*, and Δ*MoAE4*/*MoAE4* following treatment with 2.5 or 5 mM H_2_O_2_. (**C**) DAB staining of leaf sheath cells of rice infected by wild type, mutant and complementation strains at 36 hpi. Scale bar = 10 μm. (**D**) Statistical analysis of DAB staining of leaf sheath cells infected by different strains. (**E**) Endogenous H_2_O_2_ assay. The strains of hyphae of Endogenous H_2_O_2_ were determined as described in Experimental Procedures. The above experiments were performed in triplicate and repeated three independent times for each strain. Error bars represent the ± SD of three independently repeated samples, ns *p* > 0.05. ** *p* < 0.01.

To address the relationship between *MoAE4* and endogenous H_2_O_2_, the wild type and mutant strains were cultured on CM agar supplemented with 2.5 or 5 mM H_2_O_2_ at 28 °C for 5 days. As a result, Δ*MoAE4* was markedly inhibited in mycelial growth under H_2_O_2_ stress, indicating a role of *MoAE4* in oxidative stress tolerance (Figure 7A,B). As rice plant accumulates more H_2_O_2_ during pathogen-rice interaction, and *MoAE4* expression increases with pathogenic development of *M. oryzae*, we speculate that MoAE4 is responsible for the clearance of host-derived H_2_O_2_ during infection. To test this, DAB staining was used to identify the endogenous ROS accumulated in the cells of rice leaf sheath infected by *M. oryzae* at 36 hpi (Figure 7C). In the leaf sheaths inoculated with the Δ*MoAE4* strains, more than 60% of the infected cells investigated were stained dark brown; in contrast, less than 20% of the infected cells were stained light brown or colorless as Δ*MoAE4*/*MoAE4* and wild type (Figure 7C,D), displaying loss of H_2_O_2_ scavenging function in Δ*MoAE4*. Additionally, endogenous H_2_O_2_ was measured in *M. oryzae* more H_2_O_2_ accumulated in Δ*MoAE4* than in the wild type and Δ*MoAE4*/*MoAE4* (Figure 7D). These results reveal that *MoAE4* is responsible for regulating H_2_O_2_ levels exogenous, endogenous, or plant-derived.

## 4. Discussion

The family of bicarbonate transport proteins are involved in a wide-range of physiological processes in humans and mammals [1,2,42]. Mutation or dysregulation of these transporters results in physiological diseases in humans [43]. Therefore, bicarbonate transporters have attracted medical attention and have been extensively studied. In fungi, such as the unicellular organism yeast, CO_2_ can diffuse directly out of yeast cells, so the anion exchanger/carbonic anhydrase system and HCO_3_^−^ transporter are proposed to be dispensable [32]. Indeed, the AE1 homologue, YNL275w has been verified as an anion transporter just serving for boron detoxification or tolerance in *S. cerevisiae* [12,13,14]. Bicarbonate gradients modulate growth and colony morphology in *A. nidulans* [44]. Bioinformatically, other filamentous fungi harbor homologous of mammal HCO_3_^−^ transporters. In *A. nidulans*, there are at least five members of SLC family, but the most homologous *SbtB* still showed boron transporter, not HCO_3_^−^ transporter [16]. Considering the exclusive activity of BOR1 in fungi and plants, YNL275w and homologues should be grouped into the secondary bicarbonate transporter family specific for boron. The *bona fide* HCO_3_^−^ transport function in fungal YNL275w homologues is actually unknown. In this research, we demonstrated that the MoAE4, as a YNL275w homologue, plays a role in HCO_3_^−^ transport. Importantly, MoAE4 located to cytomembrane and tonoplast promotes conidiation, appressorium formation, and pathogenesis in *M. oryzae*.

In general, AEs localize to cytoplasmic membrane [10,35]. GFP-tagged Bor1p were detected to localize preferentially to the vacuole and that cells lacking Bor1p have fragmented vacuoles [45]. Recently, kidney anion exchanger 1(kAE1) has been detected on vacuole [46]. These findings suggest Bor1p functions on both cytomembrane and tonoplast. Additionally, in our study, MoAE4 was localized on cytomembrane and tonoplast (Figure 2A; Table S1). In hyphae, MoAE4 protein was concentrated on tonoplast; but in conidia, a strong fluorescence signal was on cytomembrane, suggesting the different subcellular patterns of MoAE4. This may reflect the specific function of MoAE4 in different developmental stages of *M. oryzae*.

In addition to the homology with BOR1 in sequences and 3-D structures (Figure 1), MoAE4 rescued the defect of Δ*ScBOR1* in boron tolerance, suggesting an authentic BOR1 homologue. However, MoAE4 also endowed the NaHCO_3_ tolerance for the mutant strains, for even the wild type failed to grow normally under NaHCO_3_ stress (Figure 2C). Particularly, loss of *MoAE4* resulted in the severe mycelial growth inhibition in Δ*MoAE4* compared with the wild type and complementary strains (Figure 3A,B), implying the HCO_3_^−^ transport activity in MoAE4. Based on the lucigenin fluorescence quenching assay, a great amount of HCO_3_^−^ was detected in the cytosol of Δ*MoAE4*, but not in vacuoles of Δ*MoAE4*; on the contrary, HCO_3_^−^ was only detected in vacuole in the wild type and Δ*MoAE4*/*MoAE4* (Figure 4), revealing the MoAE4-associated bicarbonate resistant mechanism, by which MoAE4 removes redundant HCO_3_^−^ from cytosol to vacuole and outside cells.

As a pathogenic fungus, conidiation and appressorium formation are key processes for disease cycle and infection [17,18]. MoAE4 loss resulted in the impaired conidiophore formation and then decreased conidial and appressorial productivity (Figure 5). We speculate that this may be related to the excessively accumulated H_2_O_2_ in the MoAE4 deletion mutant (Figure 7D). After all, Δ*MoAE4* became sensitive under H_2_O_2_ stress (Figure 7B,C). At this point, the reduced pathogenicity in Δ*MoAE4* could also be partially explained because Δ*MoAE4* was exposed to a high hydrogen peroxide stress both in vivo and in vitro (Figure 7A,E and Figure S6).

**Figure 8:**
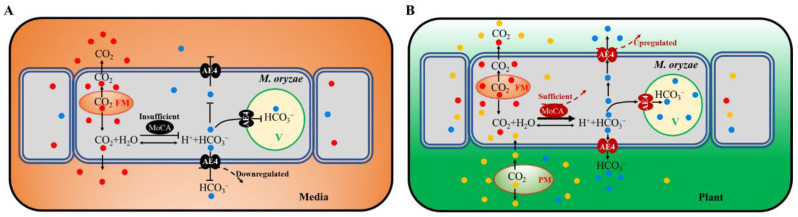
A pathogenic model mediated by MoAE4/MoCA (carbonic anhydrase) system. (**A**) *M. oryzae* is under cultivation conditions. (**B**) *M. oryzae* infects the host cells. FM, Fungal Mitochondrion; PM, Plant mitochondrion; V, vacuole; MoCA, *M. oryzae* carbonic anhydrase.

CO_2_, as a labile molecule, is the oxidation waste product of mitochondrial respiration. In humans, redundant CO_2_ must be released, or the equilibrium with HCO_3_^−^ + H^+^ will be disturbed. The ability of HCO_3_^−^ to undergo pH-dependent conversions is central to its physiological role [1]. CO_2_ enters the cytoplasm through the membrane and is rapidly hydrated forming carbonic acid (H_2_CO_3_). This acid is dissociated into H^+^ and HCO_3_^−^ by intracellular carbonic anhydrases [7]. Under normal medium culture conditions, pathogenic fungi such as yeast may not require the anion exchanger/carbonic anhydrase system to help the release of metabolic CO_2_ [11]. However, the interaction system between host plant and pathogen forms a whole multicellular organism, which should face a challenge in moving membrane impermeant bicarbonate from inside the cell where it is produced to the environment for disposal. In addition, during interactions between plant and pathogen, respiration from both pathogen and plant must be enhanced to produce available energy [24,47,48,49]. Therefore, we propose a pathogenic model mediated by MoAE4/MoCA (carbonic anhydrase) system (Figure 8). Under cultivation conditions, the metabolic CO_2_ can be released freely from *M. oryzae*, and both *MoAE4* and *MoCA* genes are at low levels of expression because of the equilibrium of (CO_2_ + H_2_O ⇌ HCO_3_^−^ + H^+^) (Figure 8A). In the process of invasive hyphae growth, the infected plant cell is a relatively high concentration of CO_2_ and low concentration of O_2_ microenvironment; and accordingly, the diffusion of fungal CO_2_ to the outside (cytosol of plant cell) is hindered. The upregulated MoCA (Figure S5) will increase the concentration of HCO_3_^−^, which leads to MoAE4 being upregulated, then MoAE4 transports HCO_3_^−^ to the vacuole or to plant cells (Figure 8B).

In the model, MoAE4 maintains the homeostasis of intracellular CO_2_-HCO_3_^−^ system, which further ensures the intracellular acid-base balance in cells [50]. According to the importance of the acid-base equilibrium in multiple physiological activities [51], we imply HCO_3_^−^ regulated by MoAE4 is a pathogenic signal for blast disease development. Actually, in our recent research, a low dose of sodium carbonate can induce the expression of a large number of disease-related genes in wild type, not in the *MoAE4* deletion mutant (unpublished data), revealing a potential drug target for blast disease control. To decipher the regulation mechanism, much work remains to be completed.

## 5. Conclusions

In the rice blast fungus, MoAE4 exhibits more sequence and structure homology with AE proteins. Additionally, MoAE4, localized on cytomembrane and tonoplast, possesses boron and NaHCO_3_ tolerance in yeast. Lucigenin fluorescence quenching assay indicated that MoAE4 has HCO_3_^−^ transport activity. Meanwhile, Δ*MoAE4* contained more H_2_O_2_ than the wild type and complementary strains did, implying a role of MoAE4 in energy metabolism. Importantly, MoAE4 is involved in conidiation, appressorium formation, and pathogenicity in *M. oryzae*. Overall, MoAE4, as a cytomembrane and tonoplast HCO_3_^−^ transporter, promotes pathogenesis of *M. oryzae*. Based on these results, a pathogenic model mediated by MoAE4 is proposed.

## Supplementary Materials

The following are available online at https://www.mdpi.com/article/10.3390/jof7110955/s1, Figure S1. Sequence alignment and prediction of MoAE4 structure. Figure S2. The construction strategies for MoAE4 deletion and complementation strains. Figure S3. The green fluorescence signals detection of intracellular pH in conidia. Figure S4. The loss of the *MoAE4* gene has a negative effect on sporulation, appressorial development. Figure S5. Transcription abundance of *MoCA* during disease development. Figure S6. Standard curve line and the endogenous H_2_O_2_ of strains hyphae. Table S1. Identifying sub-cellular location. Table S2. Primers used in this study.
