# Supplementary material for: The Bicarbonate Transporter (MoAE4) Localized on Both Cytomembrane and Tonoplast Promotes Pathogenesis in Magnaporthe oryzae
Source: J Fungi (Basel). 2021 Nov 11;7(11):955. doi: 10.3390/jof7110955 (PMC8624833; doi:10.3390/jof7110955)
Supplement: Supplementary file 1 [file jof-07-00955-s001.zip › jof-1451024-supplementary.pdf]

Table S1. Identifying sub-cellular location

Location weights	LocDB	PotLocDB	Neural Nets	Pentamers	Integral
Vacuolar	10.0	0.0	0.00	1.51	9.89
Plasma membrane	0.0	0.0	2.63	0.15	0.01
Mitochondrial	0.0	0.0	0.00	0.85	0.00

Note: Integral Prediction of protein location: Membrane bound Vacuolar with score 9.9. Neural Nets -Plasma membrane with score 2.6. Pentamers- Mitochondrial with score 0.85.

Table S2. Primers used in this study

Primer name	Sequence 5'-3'	Remark
MoAE4-L-S	GGACTAGTCCGCATTTCAGTTCGTC	Amplify *MoAE4* left
MoAE4-L-A	GGGGTACC ACCCCTTGCCACTCTTC	flank sequence
MoAE4-R-S	GCTCTAGACCACACCATCTCCCTGCT	Amplify *MoAE4* right
MoAE4-R-A	CCCAAGCTTCCATCCTCCCGTCAACAC	flank sequence
MoAE4-LHYG-S	ACAACCTACCCTGAACCT	Verify *MoAE4*
MoAE4-LHYG-A	TCCATACAAGCCAACCAC	knockout mutant sequences
MoAE4-G-S	TCGGGATGGGAACGGT	Amplify *MoAE4*
MoAE4-G-A	TGGGTCGGGCGGAGGT	sequence
MoAE4-C-S	GAAGATCTATGACGGCCC ACACATCATC	Amplify *MoAE4*
MoAE4-C-A	GGACTAGTCTGTTCACCCGAGCCGACCC	complementation sequence
qRT-MoAE4-S	TGGCAGGTCGAAAAACTGGT	qRT-PCR primer of
qRT-MoAE4-A	TTCCACGCATCCTTCCAGTC	*MoAE4*
qRT-MoCA-S	AGATGAACGTCGAGGCC	qRT-PCR primer of
qRT-MoCA-A	CACCACGGAAGACAAGC	*MoCA*
HYG-S	GATGTAGGAGGGCGTGGATATGTCCT	Amplify HYG
HYG-A	AACCCGCGGTCGGCATCTACTCTATTC	sequence
Actin-S	CGTTGTTCCTATTTACGAGGG	qRT-PCR primer of
Actin-A	TTGATGTCACGGACGATTTC	*Actin*
Sc-MoAE4-S	CCCAAGCTTATGACGGCCC ACACATCATC	Amplify *MoAE4*
Sc-MoAE4-A	GGAATTCCTGTTCACCCGAGCCGACCC	complementation
Sc-MoACT-S	CGGGATCCCATGGAAGAGGAGGTCGCCG	Amplify *MoACT*
Sc-MoACT-A	GCTCTAGAGAAGCACTTGCGGTGGAC	complementation
Sc-MoACT-S	ATGTCGAATGAGAGCACACGAG	Amplify *ScBor1*
Sc-MoACT-A	TTAGTCTTCATGCTTATCACAGA	complementation

Figure S1. Sequence alignment and prediction of MoAE4 structure. A Sequence alignment. The transmembrane domain of MoAE4 was compared with *A. nidulans* SbtA gene and *S. cerevisiae* BOR1 gene. B Prediction of MoAE4 structure Protein. Possible membrane topologies of MoAE4 were predicted 10 transmembrane-spanning domains. The sequence of 87-263 was predicted as HCO_3^-_cotransport domain. EC, extracellular side; IC, intracellular side of plasma membrane. C Three-dimensional (3-D) structures of MoAE4. 3-D structures of MoAE4 were viewed from different directions.

Figure S2. The construction strategies for MoAE4 deletion and complementation strains. A Construction of pCAMBIA1303-MoAE4:: GFP. Details of the construction are described in Experimental procrdures. B The *MoAE4* gene was replaced by the hygromycin resistant cassette (HYG). To construct the replacement vector, the flanking sequences were amplified with their corresponding primer pairs and fused with the *HYG* cassette. C The strategy of Δ*MoAE4*/*MoAE4*. Details of the construction are described in Experimental procrdures. D The relative expression of *MoAE4* in strains. The wild type, deletion mutant, and complementation strains were detected via qPCR using cDNA as a template. The relative mRNA levels were calculated using the $2^{-\Delta\Delta Ct}$ method. The *Actin* was used as an internal standard reference. The data represent the means ± standard deviation (SD) of three experiments. The error bars represent the SD.

Figure S3. The green fluorescence signals detection of intracellular pH in conidia. The light (left), fluorescence confocal (middle), and merge (right) microscope observations of strains were in conidia. The green fluorescence signal of intracellular pH value of Δ*MoAE4* strains was significantly enhanced. Bar = 10 μm.

Figure S4. The loss of the *MoAE4* gene has a negative effect on sporulation, appressorial development. A Conidia formation. Conidia of the wild type, Δ*MoAE4* and Δ*MoAE4*/*MoAE4* strains from 3-day-old mycelium grown on oatmeal agar were transferred to cover slips, induced for 6, 12, 24, and 48 h, and observed and counted under a light microscope at room temperature. Bar=50 μm. B Observation of conidial germination. Conidial germination was observed on a hydrophobic cover slips under the microscope at 1, 2, and 3 hours post inoculation. Bars = 20 μm. C Observation of the formation of appressoria. Appressorial formation was observed on a hydrophobic cover slips under the microscope at 4 and 6 hours per inoculation. Bars = 20 μm.

Figure S5. Transcription abundance of *MoCA* during disease development.

Figure S6. Standard curve line and the endogenous H_2O_2 of strains hyphae. A Standard curve line of absorbance and hydrogen peroxide content. R^2=0.9955. B DAB staining of hyphae of the wild type, mutant and complementation strains.

Fig S1.

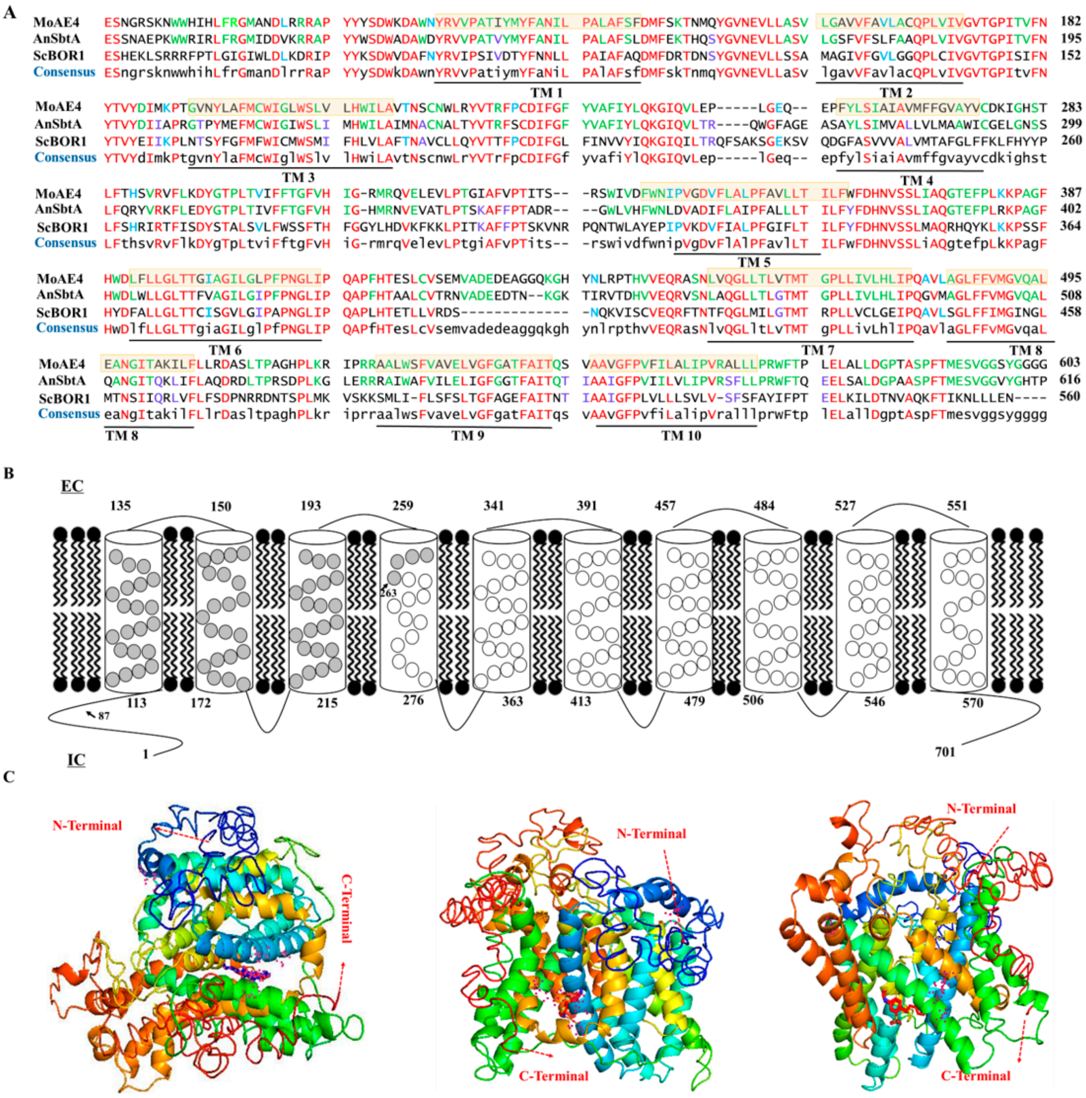
A
MoAE4
AnSbtA
ScBOR1
Consensus
TM 1
TM 2
TM 3
TM 4
TM 5
TM 6
TM 7
TM 8
TM 9
TM 10
B
EC
IC
135
150
193
259
341
391
457
484
527
551
113
172
215
276
363
413
479
506
546
570
87
263
1
701
C
N-Terminal
C-Terminal

Fig S2.

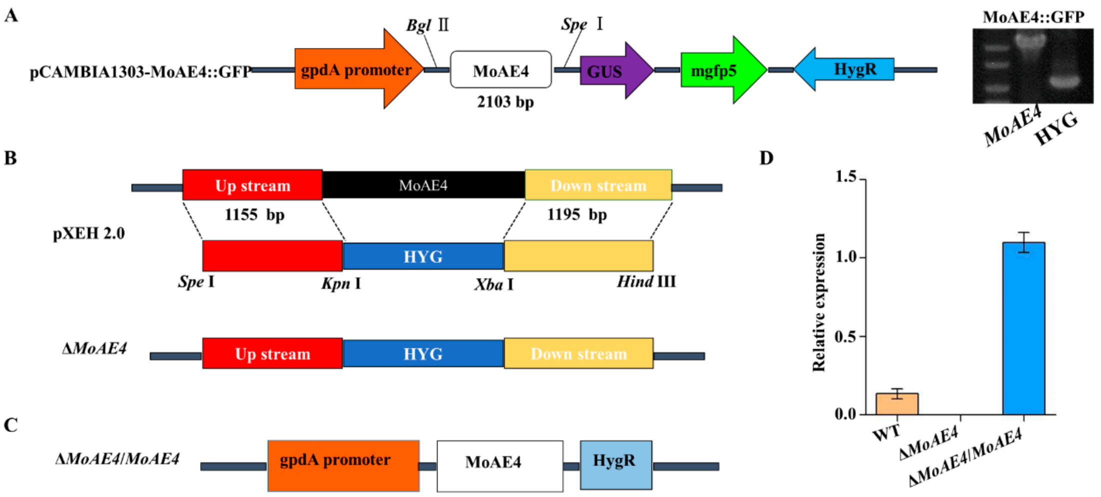

A
pCAMBIA1303-MoAE4::GFP
gpdA promoter
Bgl II
MoAE4
2103 bp
Spe I
GUS
mgfp5
HygR
MoAE4::GFP
MoAE4
HYG
B
Up stream
MoAE4
Down stream
1155 bp
1195 bp
pXEH 2.0
HYG
Spe I
Kpn I
Xba I
Hind III
ΔMoAE4
Up stream
HYG
Down stream
C
ΔMoAE4/MoAE4
gpdA promoter
MoAE4
HygR
D
Relative expression
1.5
1.0
0.5
0.0
WT
ΔMoAE4
ΔMoAE4/MoAE4

Fig S3.

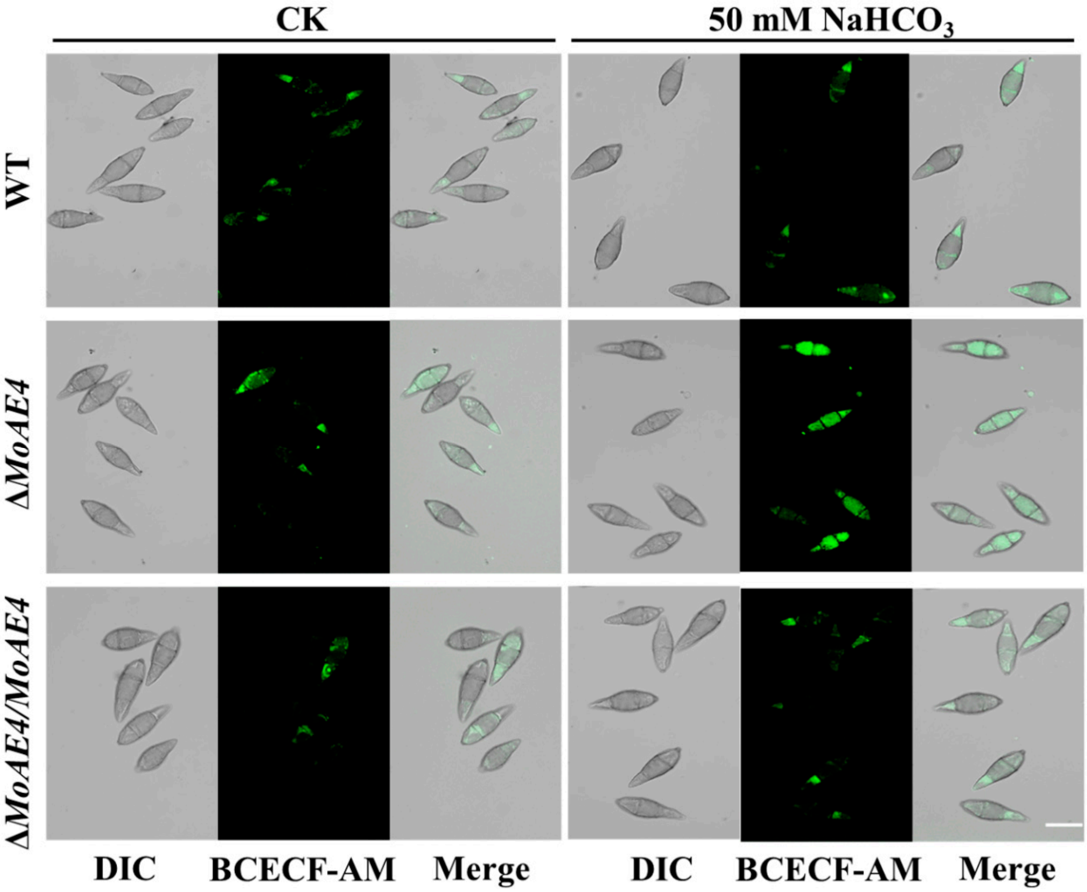

CK
50 mM $NaHCO_3$
WT
ΔMoAE4
ΔMoAE4/MoAE4
DIC
BCECF-AM
Merge
DIC
BCECF-AM
Merge

Fig S4.

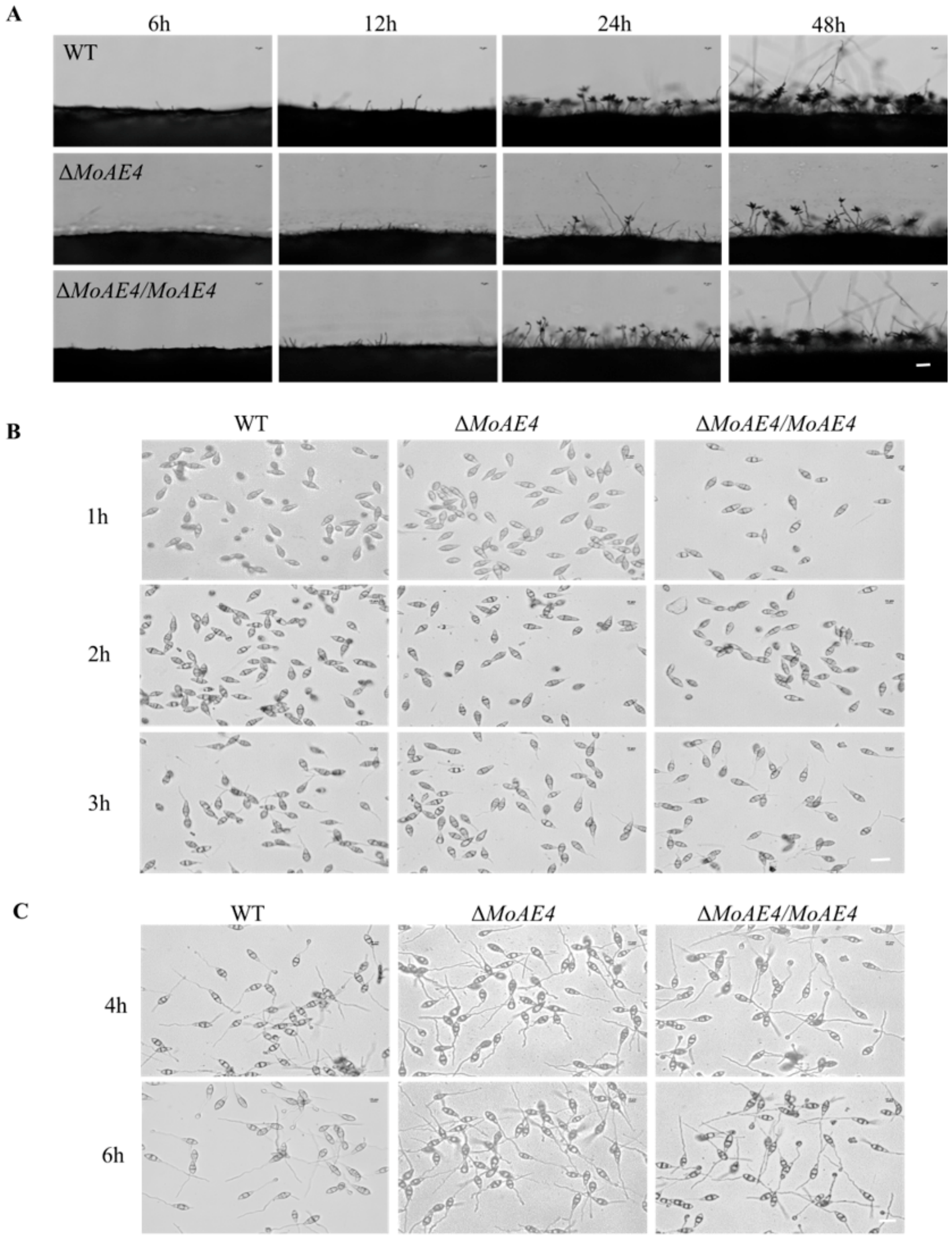
A
6h
12h
24h
48h
WT
ΔMoAE4
ΔMoAE4/MoAE4
B
WT
ΔMoAE4
ΔMoAE4/MoAE4
1h
2h
3h
C
WT
ΔMoAE4
ΔMoAE4/MoAE4
4h
6h

Fig S5.

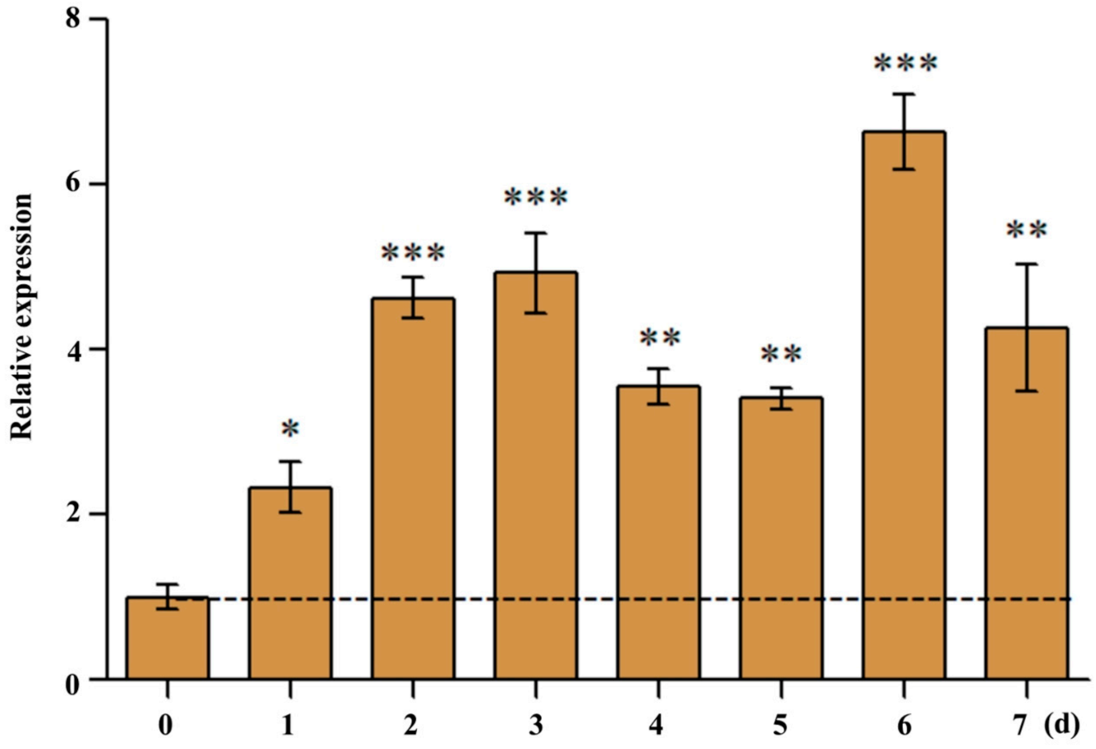

Fig S6.

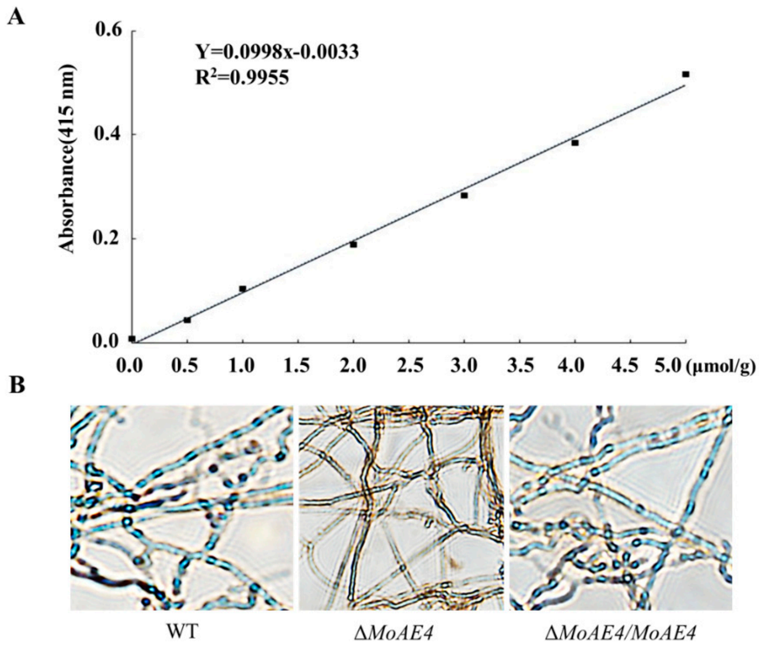
A
Y=0.0998x-0.0033
R2=0.9955
Absorbance(415 nm)
0.6
0.4
0.2
0.0
0.0 0.5 1.0 1.5 2.0 2.5 3.0 3.5 4.0 4.5 5.0 (μmol/g)
B
WT
ΔMoAE4
ΔMoAE4/MoAE4
